# Targeting of Endothelial Activation in Cerebral Malaria

**DOI:** 10.1371/journal.pmed.0020315

**Published:** 2005-08-23

Malaria is one of the most serious of all tropical parasitic diseases: a severe and life-threatening form of which in humans is cerebral malaria, a complication that can occur in malaria caused by Plasmodium falciparum. This grave complication involves malarial infection of the red blood cells that accumulate within the very small capillaries that flow through the tissues of the brain. Even when treated, cerebral malaria has a fatality rate of 15% or more.

Numerous studies have pointed to a key role of tumor necrosis factor (TNF) and related proteins in the pathogenesis of cerebral malaria, and a clear relationship has been established between plasma concentrations of TNF and cerebral pathology. In experimental cerebral malaria, TNF-beta, now called lymphotoxin α (LT), has been shown to be a principal mediator of pathogenesis. LT and TNF belong to the same family, interact with a common receptor, and could act together during disease progression to effect the pathogenesis, according to recent evidence.

Now Georges Grau and colleagues describe the anti-inflammatory activity of a transcriptional inhibitor of TNF, called LMP-420, which might offer a new way for treating cerebral malaria. The aim of their study was to assess the ability of LMP-420 to inhibit the in vitro TNF and/or LT effects on brain endothelium, with particular attention to endothelial cell activation, adhesiveness for malarial parasites, and vesiculation.

Using an in vitro model of cerebral malaria based on human, brain-derived endothelial cells (HBEC-5i), they found that LMP-420 potently reduced endothelial activation, endothelial adhesiveness for P. falciparum–parasitized red blood cells, and endothelial MP release, three major features of cerebral malaria.

The results provide evidence for a dual inhibitory effect of LMP-420 on both TNF and LT in an in vitro model of cerebral malaria pathogenesis, when added either before or simultaneously with both cytokines, they said. LMP-420 also abolished the cytoadherence of ICAM-1-specific P. falciparum–parasitized red blood cells on these endothelial cells. Identical but weaker effects were observed when LMP-420 was added with LT. LMP-420 also caused a dramatic reduction of HBEC-5i vesiculation induced by TNF or LT stimulation.

Several molecules inhibiting TNF, such as monoclonal antibody to TNF or pentoxyfylline, have been tested in clinical trials of cerebral malaria but failed to improve disease outcome. These failures could be explained by the fact that LT was recently demonstrated to also have a crucial role in the pathogenesis of this cerebral syndrome, said the authors.

They conclude that the anti-inflammatory activity of LMP-420 might be useful in targeting the wide variety of diseases in which TNF and its related family members play a role, and could represent a novel, stable, and efficient therapeutic way to improve the outcome of patients with cerebral malaria. [Fig pmed-0020315-g001]

**Figure pmed-0020315-g001:**
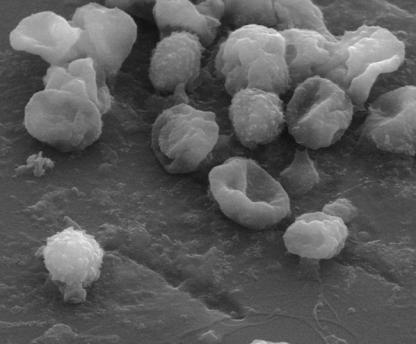
Plasmodium-infected red blood cells bind to brain endothelial cells

However, the authors caution that the experimental in vitro results do not necessarily predict potential efficacy in either animal models or humans, especially since in their model the LMP-420 was administered before the disease process was established. Nevertheless, this avenue is a promising one to explore further.

